# Detection of sarcopenia using deep learning-based artificial intelligence body part measure system (AIBMS)

**DOI:** 10.3389/fphys.2023.1092352

**Published:** 2023-01-26

**Authors:** Shangzhi Gu, Lixue Wang, Rong Han, Xiaohong Liu, Yizhe Wang, Ting Chen, Zhuozhao Zheng

**Affiliations:** ^1^ Department of Computer Science and Technology, Institute for Artificial Intelligence, and BNRist, Tsinghua University, Beijing, China; ^2^ School of Medicine, Tsinghua University, Beijing, China; ^3^ Department of Radiology, Beijing Tsinghua Changgung Hospital, School of Clinical Medicine, Tsinghua University, Beijing, China; ^4^ Department of Bioinformatics, Fujian Key Laboratory of Medical Bioinformatics, School of Medical Technology and Engineering, Fujian Medical University, Fuzhou, China

**Keywords:** abdomen, segmentation, artificial intelligence, sarcopenia, deep learning

## Abstract

**Background:** Sarcopenia is an aging syndrome that increases the risks of various adverse outcomes, including falls, fractures, physical disability, and death. Sarcopenia can be diagnosed through medical images-based body part analysis, which requires laborious and time-consuming outlining of irregular contours of abdominal body parts. Therefore, it is critical to develop an efficient computational method for automatically segmenting body parts and predicting diseases.

**Methods:** In this study, we designed an Artificial Intelligence Body Part Measure System (AIBMS) based on deep learning to automate body parts segmentation from abdominal CT scans and quantification of body part areas and volumes. The system was developed using three network models, including SEG-NET, U-NET, and Attention U-NET, and trained on abdominal CT plain scan data.

**Results:** This segmentation model was evaluated using multi-device developmental and independent test datasets and demonstrated a high level of accuracy with over 0.9 DSC score in segment body parts. Based on the characteristics of the three network models, we gave recommendations for the appropriate model selection in various clinical scenarios. We constructed a sarcopenia classification model based on cutoff values (Auto SMI model), which demonstrated high accuracy in predicting sarcopenia with an AUC of 0.874. We used Youden index to optimize the Auto SMI model and found a better threshold of 40.69.

**Conclusion:** We developed an AI system to segment body parts in abdominal CT images and constructed a model based on cutoff value to achieve the prediction of sarcopenia with high accuracy.

## 1 Introduction

Sarcopenia is an aging-associated disorder that is characterized by a decline in muscle mass, strength and function. The onset of sarcopenia increases the risk of a variety of adverse outcomes, including falls, fractures, physical disability, and death (Sayer, 2010). In Oceania and Europe, the prevalence of sarcopenia ranged between 10% and 27% in the most recent meta-analysis study (Petermann‐Rocha et al., 2022). Various biomarkers for sarcopenia and related diseases have been explored on the molecular, protein, and imaging levels. Interleukin 6 (IL-6) and tumor necrosis factor alpha (TNF-α) levels may be important factors associated with frailty and sarcopenia, according to a study (Picca et al., 2022). Muscle quality was also found to be associated with subclinical coronary atherosclerosis (Lee et al., 2021). Patients diagnosed with sarcopenia are more likely to develop cardiovascular disease (CVD) than those without sarcopenia (Gao et al., 2022). These studies have provided new perspectives for a thorough comprehension of sarcopenia.

Sarcopenia can be assessed through physical examinations or self-reported SARC-F scores, but the diagnosis of sarcopenia requires multiple tests including muscle strength tests and more accurate imaging tests, in which muscle content is evaluated using either bioelectrical impedance analysis (BIA) or Dual-energy x-ray (DXA) testing. However, bioelectrical impedance is affected by the humidity of the body surface environments, which makes accurate diagnosis challenging (Horber et al., 1986). Although dual-energy x-ray testing is commonly used to evaluate sarcopenia (Shu et al., 2022), it is not yet widely available or used; even in medical institutions with DXA testing capabilities, there is a high rate of missed diagnoses of sarcopenia due to inconsistency between instrument brands (Masanés et al., 2017; Buckinx et al., 2018).

Computed tomography (CT) is considered the gold standard for non-invasive assessment of muscle quantity and quality (Beaudart et al., 2016; Cruz-Jentoft et al., 2019). Cross-sectional skeletal muscle area (SMA, cm^2^) at the level of the third lumbar vertebra (L3) is highly correlated with total body skeletal muscle mass. Adjusting SMA for height provides a measure for relative muscle mass called skeletal muscle index (SMI, cm^2^/m^2^), which is commonly used clinically as an evaluation index to determine sarcopenia. The SMI differs by gender; a study discovered that an SMI <52.4 cm^2^/m^2^ for men and <38.5 cm^2^/m^2^ for women was defined as sarcopenia (Prado et al., 2008). The calculation of SMI requires trained personnel, but the shortage of experienced health professionals hinders the practical deployment of this technology. Meanwhile, for the diagnosis of musculoskeletal disorders, a radiologist must mark a detailed outline of the body part, which, according to some studies, may take 5–6 min in a single slice of CT image, even when using a professional tool called Slice-O-Matic (Takahashi et al., 2017). To automate this laborious process, we developed a computational method that can accurately and quickly perform body part outlining and obtain quantitative measurements for clinical practice.

The field of CT-based body part analysis is expanding rapidly and shows great potential for clinical applications. CT images have been used to assess muscle tissue, visceral adipose tissue (VAT), and subcutaneous adipose tissue (SAT) compartments. In particular, CT measurements of reduced skeletal muscle mass are a hallmark of decreased survival in many patient populations (Tolonen et al., 2021). Typically, segmentation and measurement of skeletal muscle tissue, VAT, and SAT are manually performed by radiologists or semi-automatically performed at the third lumbar vertebrae (L3). However, for a wider range of abdomen such as the whole abdomen, segmentation and measurement of skeletal muscle tissue, VAT, and SAT are lacking, limiting the clinical applications of CT-based body part analysis. In this study, we focus on developing a segmentation model for a wider range of abdomen.

Deep learning applications in healthcare are undergoing rapid development (Gulshan et al., 2016; Esteva et al., 2017; Smets et al., 2021). In particular, deep learning-based technologies demonstrated great potential in medical imaging diagnosis during COVID-19 (Santosh, 2020; Mukherjee et al., 2021; Santosh and Ghosh, 2021; Santosh et al., 2022a; Santosh et al., 2022b; Mahbub et al., 2022). Recently, a number of deep learning techniques for body part analysis using CT images have been developed (Pickhardt et al., 2020; Gao et al., 2021). (Weston et al. (2018) conducted a retrospective study of automated abdominal segmentation for body part analysis using deep learning in liver cancer patients. Zhou et al. (Zhou et al. (2020) developed a deep learning pipeline for segmenting vertebral bodies using quantitative water-fat MRI. Pickhardt et al. (Kalinkovich and Livshits, 2017) developed an automated CT-based algorithm with pre-defined metrics for quantifying aortic calcification, muscle density, and visceral/subcutaneous fat for cancer screening. (Grainger et al. (2021) developed a deep learning-based algorithm using the U-NET architecture to measure abdominal fat on CT images.

Previous studies were often limited to two-dimensional (2D) analysis of body composition using the L3 levels and did not extend to the three-dimensional (3D) abdominal volume levels. Compared with the L3-level 2D information, the 3D abdominal information is more informative and may be better associated with certain diseases. Therefore, there is a clinical need for such a segmentation tool, which is capable of performing both L3 single-level and even the whole volume of 3D abdominal CT segmentation. In comparison to previous studies, this paper focuses exclusively on automatic body part segmentation using deep learning and exploring the feasibility of predicting sarcopenia.

## 2 Materials and methods

### 2.1 Study populations

#### 2.1.1 Developmental dataset

We retrospectively analyzed patients who underwent abdominal CT plain scan examinations at the Department of Diagnostic Radiology, Tsinghua ChangGung Hospital, Beijing, China, between January 2020 and December 2020. Inclusion criteria included the following patient information: a) complete demographic information, including age and gender; b) abdominal CT plain scan examination with a scan range from the top of the diaphragm to the inferior border of the pubic symphysis; and c) absence of major abdominal diseases. Exclusion criteria included poor-quality abdominal CT scan images and noticeable artifacts that interfered with the identification of body parts. As a result, we obtained a “segmentation developmental dataset” consisting of 5,583 slides from 60 cases, 45 males and 15 females with a mean age of 32.0 ± 6.6 years (20–57 years).

#### 2.1.2 Independent test dataset

We retrospectively selected and analyzed female patients who underwent abdominal CT plain scan examinations at the Department of Diagnostic Radiology, Beijing Tsinghua Changgung Hospital, Beijing, China, between November 2014 and May 2021. In addition to the aforementioned inclusion and exclusion criteria, female patients in post-menopause with information on age at menopause, height (m), and weight (kg) were extracted. Finally, 7 patients with a mean age of 60.1 ± 8.7 years (48–73 years) were included in the study. This dataset consists of 745 CT slides and is referred to as the “independent test dataset,” which will be used to evaluate the body part segmentation model based on abdominal CT plain scans.

#### 2.1.3 Sarcopenia prediction dataset

We retrospectively selected and analyzed female patients who underwent DXA examinations at the Department of Diagnostic Radiology, Beijing Tsinghua Changgung Hospital, Beijing, China, between November 2014 and May 2021. Inclusion criteria were: a) patient had complete demographic information, including age, age at menopause, height (m), and weight (kg); b) patient was postmenopausal; c) patient’s DXA examination included the L1-L4 vertebrae and left femoral neck; d) patient received an abdominal CT plain scan from the top of the diaphragm to the inferior border of the pubic symphysis; e) patient had no significant abdominal disease; and f) patient’s abdominal CT scan and DXA examination were taken within a 12-month interval. Exclusion criteria included the presence of metallic implants in the scan area of the DXA examination, poor image quality of abdominal CT scan, or the presence of visible artifacts that interfered with muscle identification. As a result, 330 female patients with a mean age of 68.5 ± 9.7 years (50–96 years) were included in the study and referred to as the “Sarcopenia prediction dataset,” which will be used to evaluate the performance of the sarcopenia prediction model.

#### 2.1.4 Abdominal CT image acquisition

All CT scans in the retrospective study were obtained using either a GE Discovery 750 HD CT scanner (GE Healthcare, Waukesha, Wisconsin, United States of America) or an uCT 760 CT scanner (United-Imaging Healthcare, Shanghai, China). All scans were acquired in the supine position. The parameters of the CT scan were as follows: 120 kVp, auto-mAs, slice thickness: 5 mm, Pitch 1.375 mm (GE Discovery 750 HD)/0.9875 mm (uCT 760), and 512 × 512 matrix size.

#### 2.1.5 Sarcopenia diagnosis

Radiologists selected an L3 layer abdominal CT image and used ITK-SNAP software to outline the muscle region and calculate its area. Then, Eq. 1 in the following was used to calculate the skeletal muscle index (SMI). Patients with an SMI value lower than a specific threshold will be diagnosed with sarcopenia.

$$SMI\left({cm}^{2}/m^{2}\right)=\frac{\;Muscle\;Tissue\;Area\;\left[L3\right]\left({cm}^{2}\right)}{Square\;of\;Height\;\left(m^{2}\right)}$$ (1)

#### 2.1.6 Ethics review

The study was approved by the institutional review board and ethical committee of Beijing Tsinghua Changgung hospital. The number approved by the ethics committee is 21427-4-01.

### 2.2 AI system overview

**FIGURE 1:**
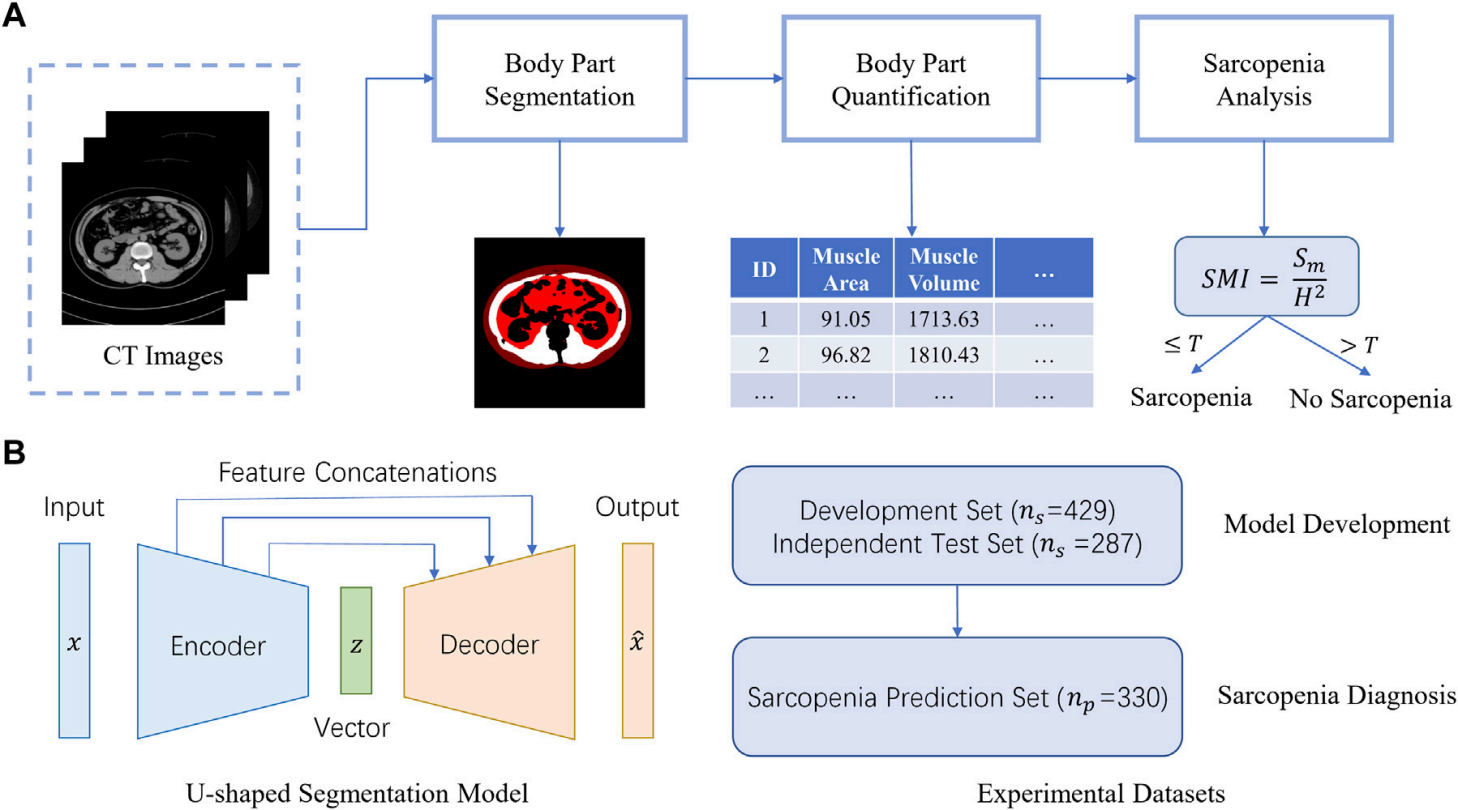
AIBMS for sarcopenia diagnosis. **(A)** Overview of the AI system. The system consists of three modules: a module for body part segmentation, a module for body part quantification, and a module for sarcopenia analysis. $S_{m}$ is the muscle tissue area, H is the height, and T is the threshold of the SMI. **(B)** U-shape encoder-decoder structure (left) and experimental datasets (right).

Figure 1A gives an overview of the Artificial Intelligence Body Part Measure System (AIBMS) for sarcopenia diagnosis. The system consists of three modules: a module for body part segmentation, a module for body part quantification, and a module for sarcopenia analysis. Using each CT scan as input, the body part segmentation module identifies areas for muscle tissue, visceral adipose tissue, and subcutaneous adipose tissue. The body part quantification module then calculates the areas and volumes of these body parts. The sarcopenia analysis module uses the 2D areas or 3D volumes for sarcopenia prediction. The SMI value at the L3 layer is calculated using the muscle tissue area ( $S_{m}$ ) and the height ( H ), and sarcopenia is diagnosed if the SMI value is greater than the threshold ( T ). The details of each module are described in the following.

### 2.3 The body part segmentation module

#### 2.3.1 Datasets

For automatic abdominal body part segmentation, CT images from 60 patients in the developmental dataset were used to develop a deep learning segmentation model. For each patient, we extracted the abdomen area by truncating the top 10% and bottom 30% of the CT scans. We then took the L4 layer images, which is commonly used in the abdominal disease identification, and randomly selected 10% out of the remaining 60% images, resulting in a set of 429 CT images. A physician with 8 years of experience in diagnostic abdominal imaging manually segmented subcutaneous fat, visceral fat, and muscle tissues. These 429 CT images were randomly divided into training, validation, and test sets with an 8:1:1 ratio at the patient level. In addition, the body part segmentation performance of this segmentation model was evaluated using the independent test dataset comprised of abdominal CT images from 7 patients.

#### 2.3.2 Network architecture

The U-shaped encoder-decoder structure (Figure 1B, left) was used to construct a segmentation model. The encoder network takes a CT scan as input to extract scan features ranging from low-levels such as individual pixels, to high levels such as body parts. Then the decoder network expands high-level features back to low level features to produce the pixel-level contour and area for each body part, which is known as “a segmentation map”. There are feature concatenations between the corresponding layers of the encoder and the decoder. To train the network, the binary cross entropy loss was used as an objective function for the pixel-level binary classification task. In this study, we adopted the following two classic U-shaped encoder-decoder deep learning models: U-NET (Ronneberger et al., 2015), Attention U-NET (Oktay et al., 2018). And we also used SEG-NET for comparison (Badrinarayanan et al., 2015).

For U-NET, the encoder’s contracting path contains four identical blocks using the standard convolutional network architecture. Each block comprises of two 3 × 3 convolutions (unpadded) followed by a rectified linear unit (ReLU) and a 2 × 2 max pooling operation with stride 2 to reduce the size of the feature map by half (downsampling). In the decoder’s expansive path, there are also four blocks, and each is parallel to one block in the encoder. Each decoder block doubles the size of the feature map (upsampling) using 2 × 2 up-convolutions and concatenates it with the feature map from the corresponding encoder block. Then it applies two 3 × 3 convolutions, each followed by a ReLU. At the final layer, a 1 × 1 convolution is employed to reduce the number of channels (features) to 3, corresponding to the segmentations of subcutaneous fat, visceral fat, and muscle tissues, respectively. Compared to U-NET, SEG-NET does not have feature concatenations, while it passes pooling indices from the encoders to the corresponding decoders, whereas Attention U-NET adds an attention gate link to the upsampling process, which allows the input features to be reweighted by their computed attention coefficients.

To train these networks, we minimize the following loss function

$$L\left(x,y\right)=L=\sum_{n=1}^{N}\sum_{k=1}^{K}l_{nk},$$ (2)

where x is the predicted mask, y is the ground truth segmentation mask, N is the batch size, K is the segmentation categories, and $l_{nk}$ is the loss of the k -th category of the n -th image. $l_{nk}$ consists of two loss functions: a binary cross entropy loss function, $l_{BCE}$ , and a soft dice loss function, $l_{DICE}$ , and $l_{nk}=l_{BCE}+l_{DICE}$ . Since we regard the prediction for each category (whether or not it belongs to a specific body part) as a binary segmentation task, the binary cross entropy loss $l_{BCE}$ and dice loss $l_{DICE}$ are shown in the following.

$$l_{BCE}=-\sum_{i=1}^{M}\left[\left.y_{i}\log p_{i}+(1-y_{i})\log(1-p_{i})\right)\right]$$ (3)

$$l_{DICE}=1-\frac{2\sum_{i=1}^{M}y_{i}*p_{i}}{\sum_{i=1}^{M}y_{i}+\sum_{i=1}^{M}p_{i}},$$

where $y_{i}$ is the ground truth of the i -th pixel belonging to certain body part, $p_{i}$ is the predicted probability of the i -th pixel $x_{i}$ of the predicted mask, $p_{i}=\sigma \left(x_{i}\right)=1/\left(1+e^{-x_{i}}\right)$ , and M is the total number of pixels of the mask.

#### 2.3.3 Deep learning settings

The segmentation models were developed on the Ubuntu 20.04 operating system. The training was conducted using a 3.80 GHz AMD® R7 5800X CPU with a GeForce GTX 1080Ti GPU. The implementation and assessment of the neural networks and statistical analysis were all carried out in the Python3.8 environment.

The segmentation network was trained, validated, and tested on the developmental dataset of 429 images from 60 cases, and then tested again on the independent test dataset. During training, we used Adam optimizer (Kingma, 2022) with four images per minibatch and set the learning rate to 1e-3. The model was trained for 100 epochs.

### 2.4 The body part quantification module

The body part segmentation performance of this segmentation model was evaluated using the independent test dataset comprised of abdominal CT images from 7 patients. The segmentation results were then used to calculate 1) the 2D abdominal body part area, as defined by the body part on the CT level passing through the middle of the L3 vertebra, and 2) the 3D abdominal body part volume, as defined by the body part on the CT level passing through the middle of the L3 vertebra, with 20 layers up and 20 layers down, for a total of 41 CT images.

#### 2.4.1 Calculation of body part volume

After calculating body part areas with the body part segmentation map, we use the areas to calculate the volume based on the following assumption. Since the portion of the body between two adjacent slices is continuous and its thickness is small, the volume between them can be approximated in the following,

$$V_{i}=\left(S_{1}+S_{2}\right)*H/2$$ (4)

where $S_{1}$ and $S_{2}$ represent the areas calculated for each of the two adjacent slices, respectively, and H represents the thickness between them, which is 5 mm in this study.

### 2.5 A quick classification model for sarcopenia based on cutoff value (Auto SMI)

In order to achieve a quick classification of sarcopenia, we segmented the L3 muscle area using the Attention U-NET-based automatic segmentation system and computed the skeletal muscle index (SMI). The SMI values with the internationally accepted cutoff value of SMI = 38.5 for females were applied to 330 patients to quickly classify sarcopenia. The results were then compared with the gold standard results by radiologists to calculate the sensitivity and specificity.

In addition, we evaluated the classification performance of this quick classification model under various cutoff values and plotted the results as an ROC curve of the Auto SMI model. We then performed the Youden Index analysis to determine the optimal threshold that maximizes the value of TPR−FPR . The coordinate point on the ROC curve corresponding to the optimal Youden index is calculated by Eq. 5.

$$index=argmax\left(TPR-FPR\right)$$ (5)

This index allows us to compute the SMI threshold.

### 2.6 Evaluation metrics and running time analysis

First, we define TP, FP, and FN as the numbers of true positive pixels that belong to the abdominal body part and are predicted by the model, false positive pixels that do not belong to the abdominal body part but are predicted incorrectly by the model, and false negative pixels that belong to the abdominal body part but are predicted incorrectly by the model, respectively. Then, we applied four metrics to evaluate the performance of the segmentation model: dice score (DSC), intersection over union (IOU), precision P), and recall R), which are defined as follows:

$$DSC=\frac{2TP}{FP+2TP+FN}$$

$$IOU=\frac{TP}{FP+TP+FN}$$

$$P=\frac{TP}{TP+FP}$$

$$R=\frac{TP}{TP+FN}$$

Using the independent test set, we counted the computational time analysis of these models in the target segmentation task of abdominal body parts. The average time to segment an image using various settings of computational resources were reported.

## 3 Results

### 3.1 Statistics of sarcopenia prediction dataset

We summarized the statistics of the training and testing datasets used for Sarcopenia prediction. As shown in Table 1, these two datasets are not statistically different in terms of age, weight, height, BMI, and prevalence of sarcopenia (*p*-value > 0.05).

**TABLE 1:** Demographic characteristics in this study.

	Training group (*n* = 264)	Testing group (*n* = 66)	*p*-value
Age (year)	68.5 ± 10.0	68.5 ± 8.4	0.98
Weight (kg)	61.6 ± 12.1	60.4 ± 9.7	0.45
Height (cm)	159.1 ± 5.9	159.4 ± 5.0	0.73
BMI (kg/m^2^)	24.3 ± 4.4	23.8 ± 3.7	0.38
Disease Status n (%)			
Sarcopenia	101 (38.3%)	27 (40.9%)	0.69
Non-Sarcopenia	163 (61.7%)	39 (59.1%)	

### 3.2 Performance of abdominal body part segmentation

The segmentation models were evaluated on the developmental test dataset and independent test dataset, obtaining DSC scores, mean IoU values, precisions, and recalls, shown in Tables 2, 3, respectively. The numbers in bold represent the best performance among the three models.

**TABLE 2:** Statistics of agreement between manual and automatic segmentations in the developmental test dataset.

	Model	U-NET	Attention U-NET	SEG-NET
Subcutaneous fat	DSC	0.978 ± 0.024	**0.981 ± 0.022**	0.802 ± 0.208
	IOU	0.956 ± 0.045	**0.962 ± 0.042**	0.681 ± 0.260
	Precision	0.973 ± 0.025	**0.976 ± 0.029**	0.693 ± 0.250
	Recall	0.983 ± 0.028	**0.985 ± 0.021**	0.965 ± 0.092
Visceral fat	DSC	0.935 ± 0.103	**0.942 ± 0.089**	0.878 ± 0.223
	IOU	0.883 ± 0.156	**0.893 ± 0.143**	0.797 ± 0.280
	Precision	0.946 ± 0.055	**0.952 ± 0.055**	0.827 ± 0.282
	Recall	0.928 ± 0.151	0.935 ± 0.132	**0.953 ± 0.072**
Muscle	DSC	0.957 ± 0.029	**0.960 ± 0.030**	0.796 ± 0.162
	IOU	0.919 ± 0.053	**0.924 ± 0.054**	0.669 ± 0.216
	Precision	**0.960 ± 0.038**	0.957 ± 0.042	0.686 ± 0.221
	Recall	0.956 ± 0.042	**0.963 ± 0.037**	0.963 ± 0.039

**TABLE 3:** Statistics of agreement between manual and automatic segmentations in the independent test dataset.

	Model	U-NET	Attention U-NET	SEG-NET
Subcutaneous fat	DSC	0.972 ± 0.047	**0.988 ± 0.021**	0.842 ± 0.088
	IOU	0.946 ± 0.083	**0.977 ± 0.041**	0.730 ± 0.131
	Precision	0.967 ± 0.042	**0.982 ± 0.033**	0.788 ± 0.145
	Recall	0.977 ± 0.069	**0.994 ± 0.021**	0.913 ± 0.135
Visceral fat	DSC	0.935 ± 0.068	**0.971 ± 0.056**	0.821 ± 0.213
	IOU	0.881 ± 0.115	**0.945 ± 0.102**	0.709 ± 0.279
	Precision	0.933 ± 0.096	**0.973 ± 0.072**	0.725 ± 0.284
	Recall	0.940 ± 0.078	0.970 ± 0.067	**0.970 ± 0.037**
Muscle	DSC	0.908 ± 0.052	**0.916 ± 0.049**	0.737 ± 0.167
	IOU	0.832 ± 0.086	**0.845 ± 0.082**	0.591 ± 0.206
	Precision	**0.902 ± 0.071**	0.894 ± 0.081	0.622 ± 0.232
	Recall	0.915 ± 0.074	**0.940 ± 0.058**	0.927 ± 0.063

**FIGURE 2:**
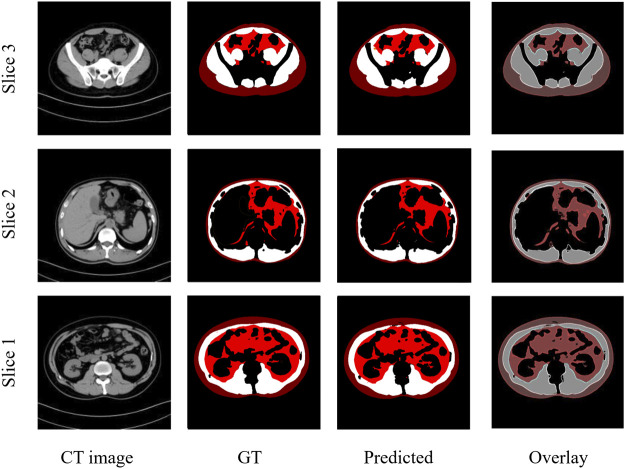
Three examples of CT images showing similar results of manual and automated segmentations. GT: Ground truth manual segmentation; Predicted: Model predicted segmentation; Overlay: Overlay of GT and Predicted.

These results demonstrate that the deep learning segmentation models perform well across both test datasets, where slides are randomly extracted either from the developmental dataset or from the 3D abdominal CT scans of the independent test dataset. Figure 2 shows three examples of the segmentation results, with the original CT images in the first column, the manual segmentations by radiologists in the second column, the automatic segmentations by the Attention U-NET model in the third column, and the overlays between the second and third columns in the fourth column. In the segmented images, subcutaneous fat is represented by dark red areas, visceral fat by bright red areas, and muscles by white areas. In the overlay pictures, the outline represents the manual segmentations.

**FIGURE 3:**
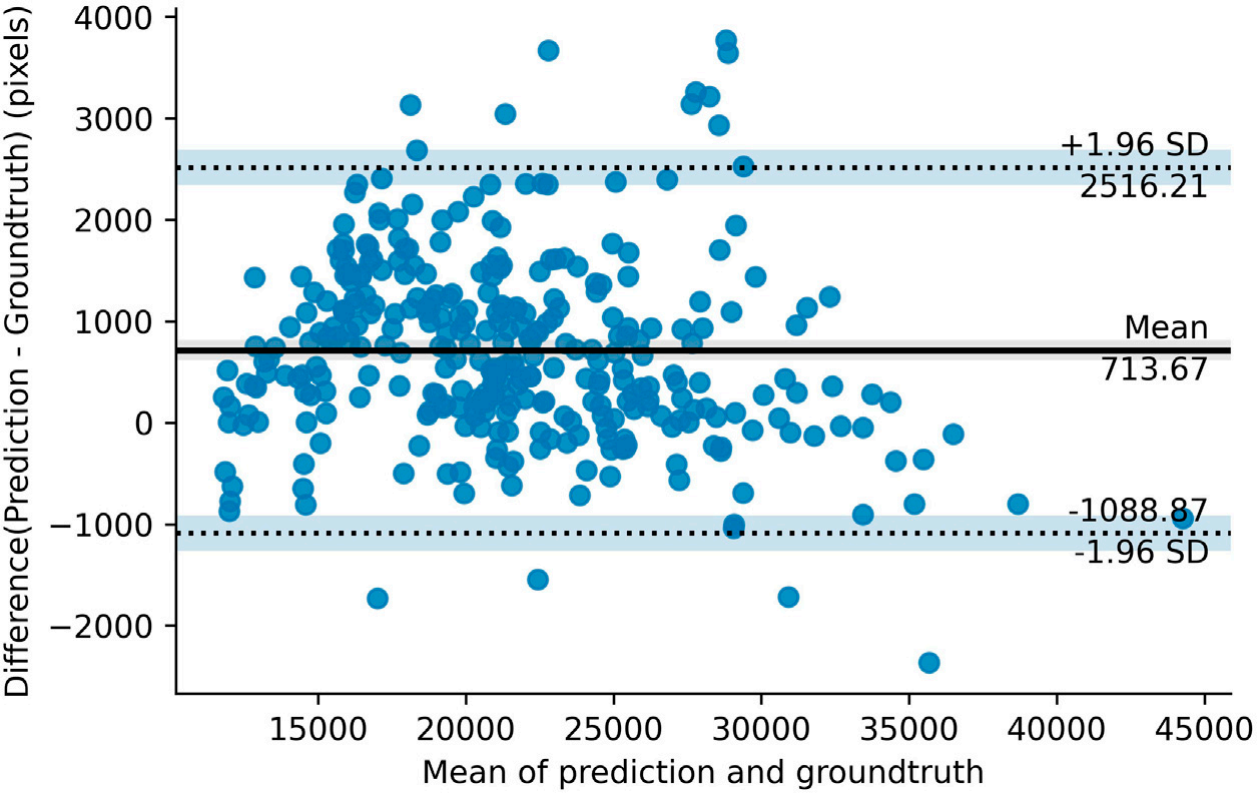
The bland-altman plot.

Figure 3 shows the Bland-Altman plot of segmented muscle regions on the test set (*n* = 333 images) using the Attention U-NET model. Each dot represents an image. The horizontal axis represents the average of the manual and automatic segmentations, as measured by the number of pixels, whereas the vertical axis represents their difference. The solid black line represents the mean difference between two segmented muscle regions, while the two dashed lines represent the 95% confidence interval (mean ±1.96*standard deviation).

### 3.3 Performance of abdominal body part volume calculation on the independent test set

Based on the segmentation results from the Attention U-NET, we calculated the volume of the abdominal body parts for each patient in the independent test set, The volumes of the subcutaneous fat, visceral fat, and muscle are shown in Tables 4–6, respectively.

**TABLE 4:** Accuracy of the subcutaneous fat volume calculation on the independent test set.

Patient ID	Ground truth	Predicted	Difference	% Of difference
1	4,461.47	4,522.96	61.49	1.36%
2	5,415.30	5,421.10	5.80	0.11%
4	3,620.08	3,639.34	19.26	0.53%
5	4,264.22	4,198.49	−65.73	−1.57%
6	2,461.13	2,473.48	12.35	0.50%
7	3,353.95	3,334.67	−19.28	−0.58%
8	3,704.44	3,702.73	−1.71	−0.05%
Mean ± sd	3,897.22 ± 933.55	3,898.97 ± 935.62	–	–

**TABLE 5:** Accuracy of the visceral fat volume calculation on the independent test set.

Patient ID	Ground truth	Predicted	Difference	% Of difference
1	3,242.54	3,251.60	9.06	0.28%
2	2,611.58	2,597.90	−13.68	−0.53%
4	3,208.96	3,254.98	46.02	1.41%
5	3,041.52	3,019.03	−22.49	−0.74%
6	772.35	789.36	17.01	2.15%
7	2,411.30	2,356.63	−54.67	−2.32%
8	865.74	861.71	−4.03	−0.47%
Mean ± sd	2,307.71 ± 1,061.78	2,304.46 ± 1,062.78	–	–

**TABLE 6:** Accuracy of the muscle volume calculation on the independent test set.

Patient ID	Ground truth	Predicted	Difference	% Of difference
1	2,317.96	2,464.54	146.58	5.95%
2	2,521.99	2,522.15	0.16	0.01%
4	1718.25	1841.16	122.91	6.68%
5	2,185.81	2,330.26	144.45	6.20%
6	1,653.20	1720.89	67.69	3.93%
7	2,233.17	2,369.07	135.90	5.74%
8	1733.22	1817.44	84.22	4.63%
Mean ± sd	2051.94 ± 345.06	2,152.22 ± 343.51	–	–

### 3.4 Running time of the deep learning models

Table 7 summarizes the time cost for processing a CT image using SEG-NET, U-NET, and Attention U-NET on a single GPU card, a single-core CPU, or a Quad-core CPU, respectively.

**TABLE 7:** Running time of deep learning models.

	GPU (Single card) s)	CPU	
		Single-core(s)	Quad-core(s)
SEG-NET	0.071	4.296	1.220
U-NET	0.074	5.861	1.872
Attention U-NET	0.077	6.039	1.886

**FIGURE 4:**
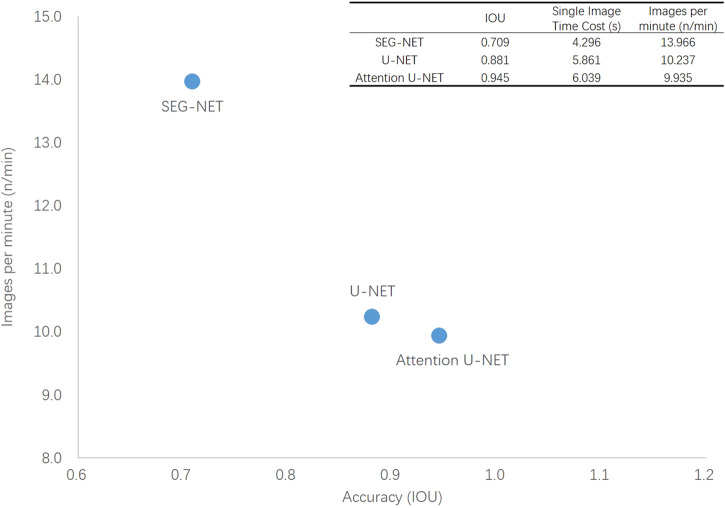
Comparison of productivity and accuracy of three deep learning models.

The results in Table 7 show that using a single-core CPU, processing a CT image using the Attention U-NET, U-NET, and SEG-NET took an average of 6.039, 5.861, and 4.296 sec, respectively. For a patient with 41 CT images, calculating the volumes of the abdominal body components takes an average of 248, 240, and 176 sec for the Attention U-NET, U-NET, and SEG-NET, respectively. Figure 4 plots the number of images per minute on a single-core CPU to better represent the model’s time utilization efficiency and the IOUs on the independent test set as a surrogate for accuracy. As can be seen, the automatic segmentation model based on the Attention U-NET is highly accurate and comparable in speed to other models. Therefore, Attention U-NET was chosen as the default model to carry out the subsequent experiments.

### 3.5 Performance of quick classification model for sarcopenia (Auto SMI)

**FIGURE 5:**
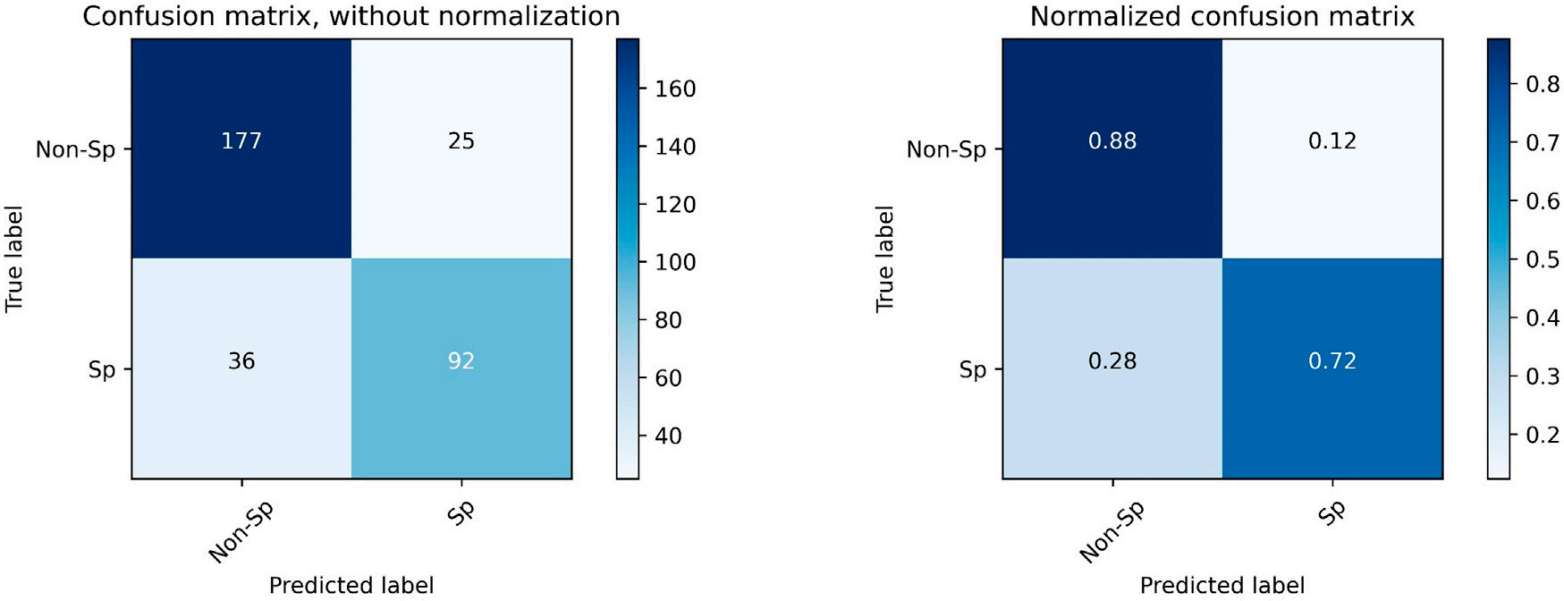
Confusion matrix for the prediction of sarcopenia with SMI = 38.5 as the cutoff value. “Sp” refers to sarcopenia, and “Non-Sp” refers to non-sarcopenia.

We applied the quick classification model to the Sarcopenia prediction dataset. The model received input from the SMI value calculated from the L3 muscle area obtained by Attention U-NET-based auto-segmentation, and SMI = 38.5 was used as the cutoff value to make sarcopenia predictions. The accuracy was 0.815, the sensitivity was 0.718, and the specificity was 0.876. Figure 5 shows the confusion matrix for the sarcopenia prediction.

**FIGURE 6:**
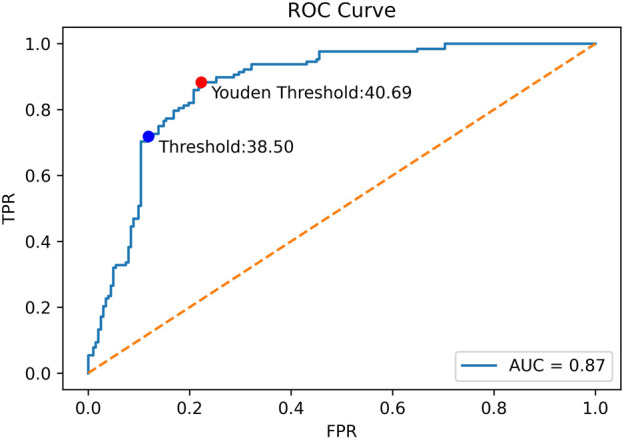
The ROC curve for quick classification model for sarcopenia prediction.

The ROC curve for the Auto SMI model is shown in Figure 6, and AUC = 0.874. In the figure, the coordinate point at the cutoff = 38.5 is denoted by a blue dot.

**FIGURE 7:**
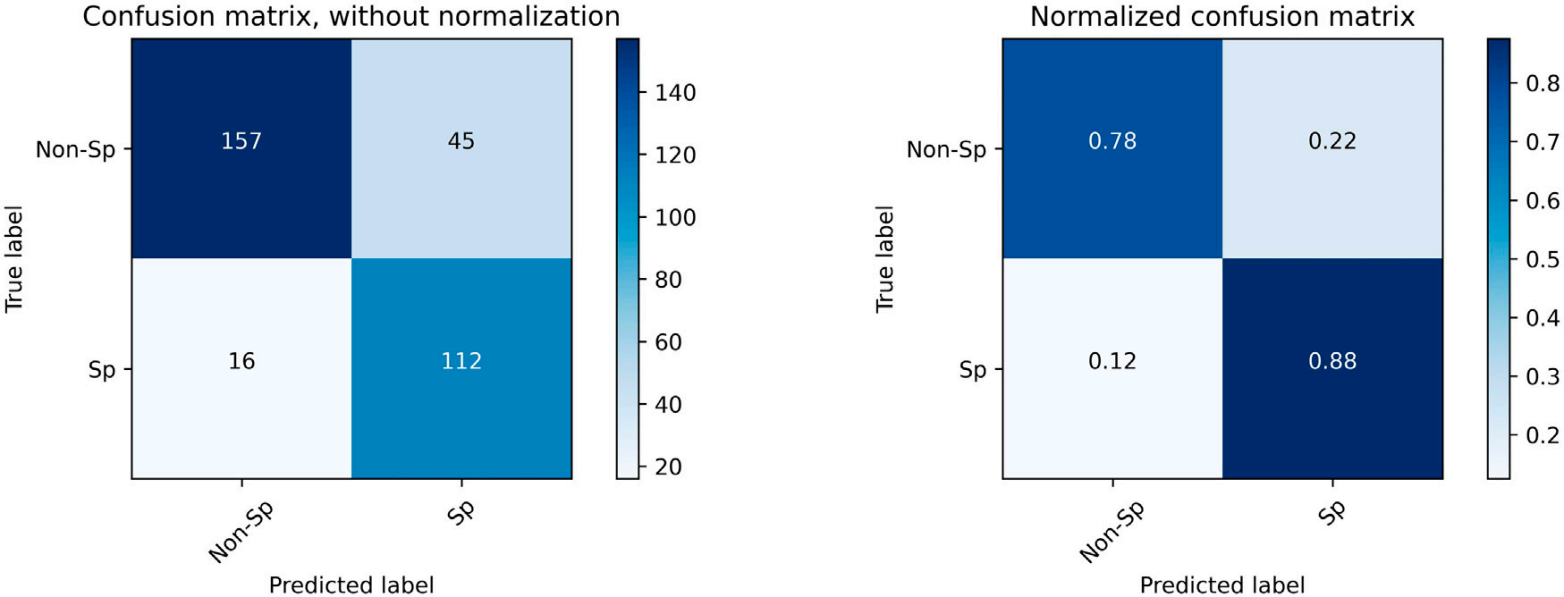
Confusion Matrix for sarcopenia prediction with SMI = 40.6 as the cutoff value. “Sp” stands for sarcopenia, and “Non-Sp” stands for non-sarcopenia.

To determine whether there is a better cutoff value for the Auto SMI, we calculated the optimal cutoff value for the Youden index, which is defined by Eq. 5. Because the Youden index is commonly used in laboratory medicine to represent the overall ability of a screening method to distinguish affected from non-affected individuals. A larger Youden index indicates better screening efficacy. Therefore, we calculated the best cutoff value (=40.69) that maximized the Youden index, indicating that the efficacy of sarcopenia screening is maximum at this cutoff value. As shown in Figure 7, the red dot represents the point with the cutoff value = 40.69.

Using a new cutoff value SMI = 40.6, the accuracy for sarcopenia prediction was 0.815, the sensitivity was 0.875, and the specificity was 0.778. Figure 7 plots the confusion matrix for the results.

### 3.6 Analysis of correlation between 2D and 3D results

**FIGURE 8:**
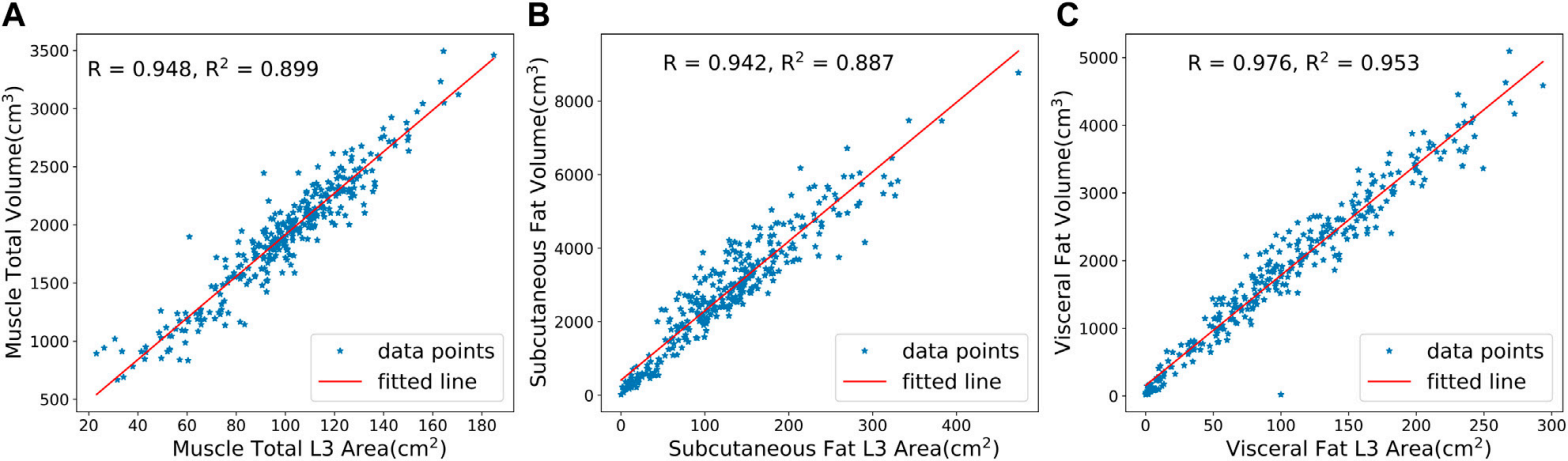
Correlation analysis between predicted 3D volumes and 2D areas at the L3 layer for **(A)** total muscle, **(B)** subcutaneous fat, and **(C)** visceral fat. R is the Pearson correlation coefficient, while R2 is the coefficient of determination.

We analyzed the correlation between the predicted 3D volume features and the 2D area features at the L3 layer using the Sarcopenia prediction dataset. Figure 8 shows a high degree of correlations between 3D features and 2D features in total muscle (*R* = 0.948), subcutaneous fat (*R* = 0.942), and visceral fat (*R* = 0.976). These results indicate the significance of the features calculated from the L3 layer. Meanwhile, the high correlation demonstrates the high accuracy of our model’s predictions in various slices.

## 4 Discussion

### 4.1 Analysis of the performance and efficiency of the models

All three models utilize an encoder-decoder structure to generate high-quality segmentation masks. SEG-NET is the simplest network with the fastest segmentation but at the cost of accuracy. U-NET uses a U-shaped structure that has been proven to perform well in medical image segmentation. It achieves better accuracy by passing the corresponding feature maps from the encoder to the decoder and fusing shallow and deep features. Attention U-NET, in comparison, adds additional Attention blocks during up-sampling, which can effectively filter out noise caused by edge polygons in labeling, thus improving performance.

In this study, we randomly selected 10% images as the developmental dataset, and we demonstrated that the deep learning segmentation model trained on the developmental dataset achieved good segmentation results on both the developmental test set and the independent test set, proving that our sampling strategy is effective. The sampling strategy has the following benefits: first, it greatly reduced the workload and time necessary for manual labeling. Second, it ensures generalizability to the entire abdominal prediction. Thirdly, this strategy reduces the workload of model training.

This design allows us to train a 2D segmentation model capable of segmenting 3D slices and generating 3D features, such as the volumes of various body parts. Compared to heavy 3D models, this 2D model is lightweight and suitable for deployment in hospitals.

With time cost analysis of the three deep learning models under different computing processors, it is evident that processing 41 images to calculate the abdominal body component volume using a CPU takes less than 1 min per patient, which is very fast. If the computing processors are GPUs, the calculation time can be shortened to a few seconds for each patient. This is based on a CT scan with a slice thickness of 5 mm; for a CT scan with a slice thickness of 0.625 mm, the volume calculation of the abdominal body components would require more computational resources, but the time cost would still be acceptable when using GPUs.

### 4.2 Characteristics of the predictive model for sarcopenia

In this study, we developed an artificial intelligence pipeline for sarcopenia prediction using abdominal body parts. Moreover, we constructed a quick classification model, the Auto SMI model, for the accurate prediction of sarcopenia. The system can be applied to patients undergoing abdominal CT scans without exposing them to additional radiation, thus enabling more efficient screening for sarcopenia.

### 4.3 Future work

Using the Artificial Intelligence Body Part Measure System (AIBMS) developed in this paper, it is possible to explore correlations between body part information generated by the system and various kinds of diseases and to provide more effective screening, auxiliary diagnosis, and even companion diagnosis. This system enables the discovery of new clinically significant biomarkers from CT images, such as the level of intramuscular fat infiltration and muscle quality, and the establishment of their correlations with diseases.

## 5 Conclusion

In conclusion, we developed an Artificial Intelligence Body Part Measure System (AIBMS) that automatically segmented and quantified the body parts in abdominal CT images, which can be used in a variety of clinical scenarios. We also developed two models to predict sarcopenia with a high accuracy.

## Data Availability

The raw data supporting the conclusions of this article will be made available by the authors, without undue reservation.
